# Successful occlusion of ventricular septal rupture in myocardial infarction under the guidance of echocardiography

**DOI:** 10.1186/s13019-019-0954-3

**Published:** 2019-07-05

**Authors:** Xiaofeng Wang, Fang Nie, Na Ye, Xuehui Liu, Shaoqing Yang, Fangzhou Guo, Jing Li

**Affiliations:** Department of Ultrasound, Lanzhou University Second Hospital, No. 80 Cuiyingmen, Chengguan District, Lanzhou City, 730030 Gansu Province China

**Keywords:** Ventricular septal rupture, Echocardiography, Interventional occlusion

## Abstract

**Introduction:**

The traditional treatment of myocardial infarction with ventricular septal rupture is surgical treatment. For the elderly patients with cardiac insufficiency, surgical treatment is very risky. The successful treatment of this case by interventional occlusion is a new method. No relevant literature reports have been found.

**Case:**

A 77-year-old man with a past medical history of old myocardial infarction presented to the physician with sudden onset of palpitation and shortness of breath. Echocardiography showed thinning of the interventricular septum near the apex and bulging toward the right ventricular side with “paradoxical motion”, on which a rupture of about 8 mm in diameter was seen. CDFI: left ventricular blood shunted to the right ventricle through the rupture.Echocardiographic diagnosis: old left ventricular anteroseptal myocardial infarction with ventricular septal rupture. Due to the older age of the patient and reduced left ventricular function, surgical repair of the ventricular septal rupture site was more difficult. After multidisciplinary discussion, it was agreed that the patient could not afford thoracotomy and was not suitable for thoracotomy, and echocardiography guided interventional occlusion of the ruptured interventricular septum could be performed.

**Conclusion:**

Transesophageal echocardiography-guided interventional occlusion of myocardial infarction with ventricular septal rupture in elderly patients with cardiac insufficiency is a new attempt, the successful treatment of this case shows that this method is feasible, for some patients is an appropriate treatment.

## Introduction

Interventricular septal rupture is a common complication of myocardial infarction and a serious threat to the lives of patients. The traditional treatment of myocardial infarction with ventricular septal rupture is surgical treatment. For the elderly patients with cardiac insufficiency, surgical treatment is very risky. The successful treatment of this case by interventional occlusion is a new method. No relevant literature reports have been found.

## Case

**Fig. 1:**
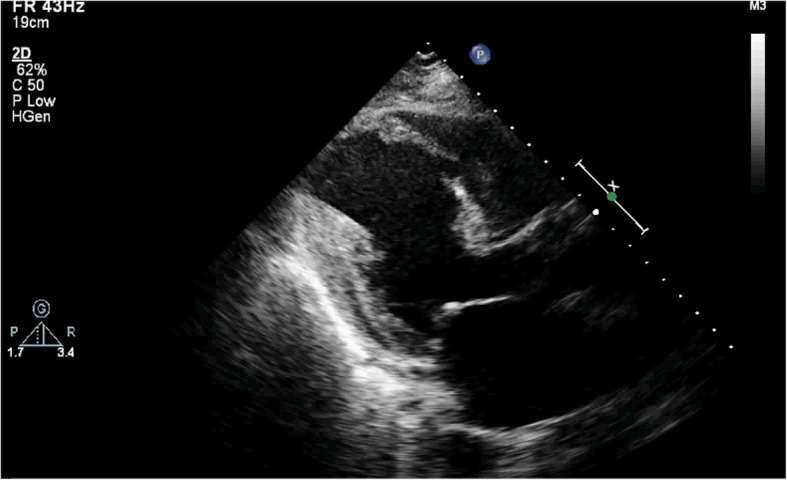
TTE left ventricular long axis view shows: the interventricular septum is thinned and bulges towards the right ventricular side with a ruptured ostium apically

**Fig. 2:**
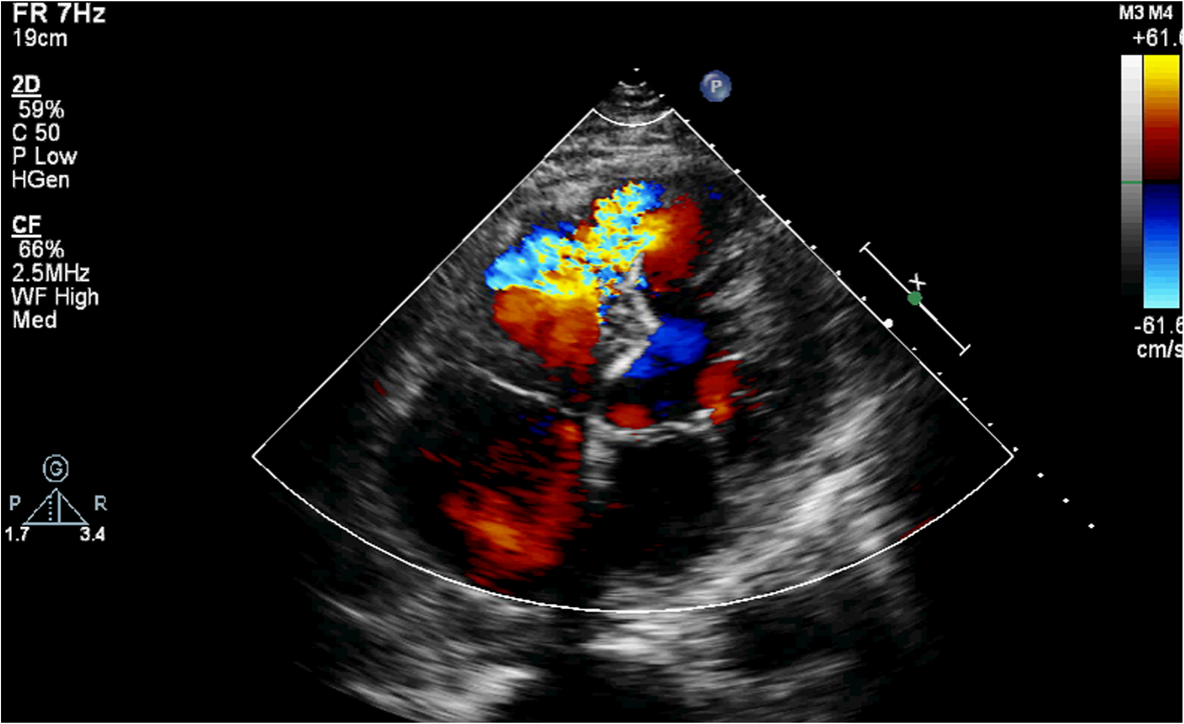
TTE apical 4-chamber view showing bright left-to-right color shunt bundle at the apex of the interventricular septum

**Fig. 3:**
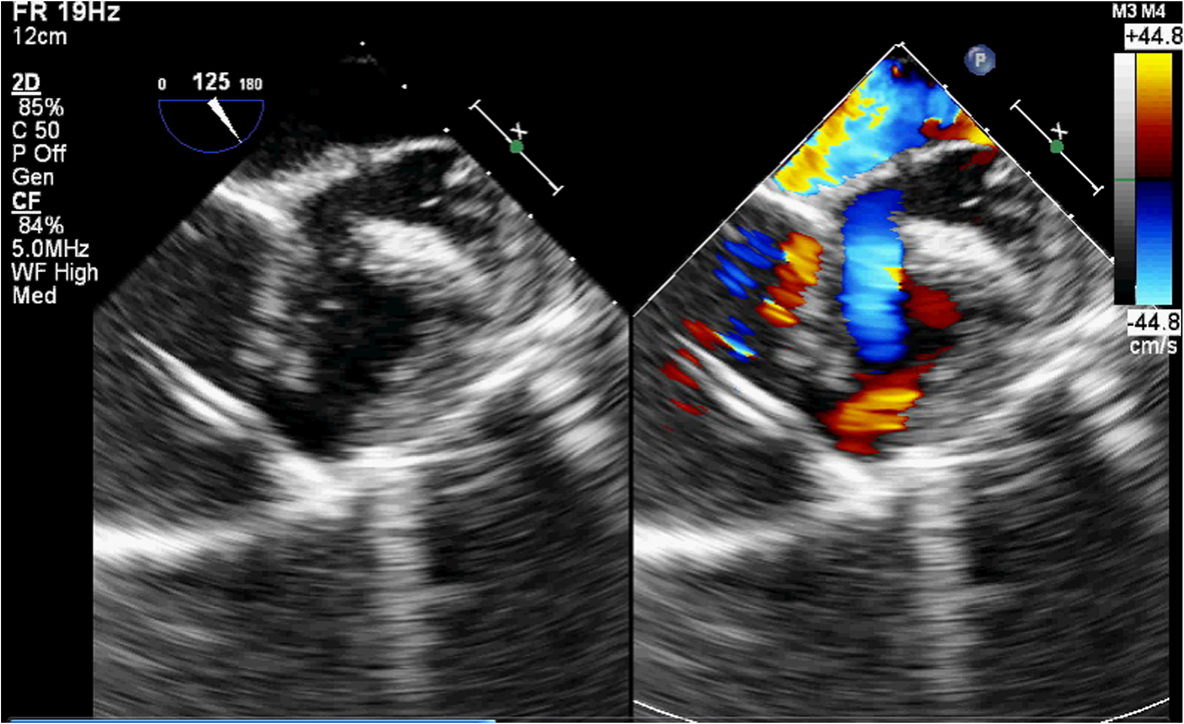
Intraoperative TEE showed that the sheath entered the heart chamber through the anterior wall of the right ventricle and entered the left ventricle through the rupture of the interventricular septum

**Fig. 4:**
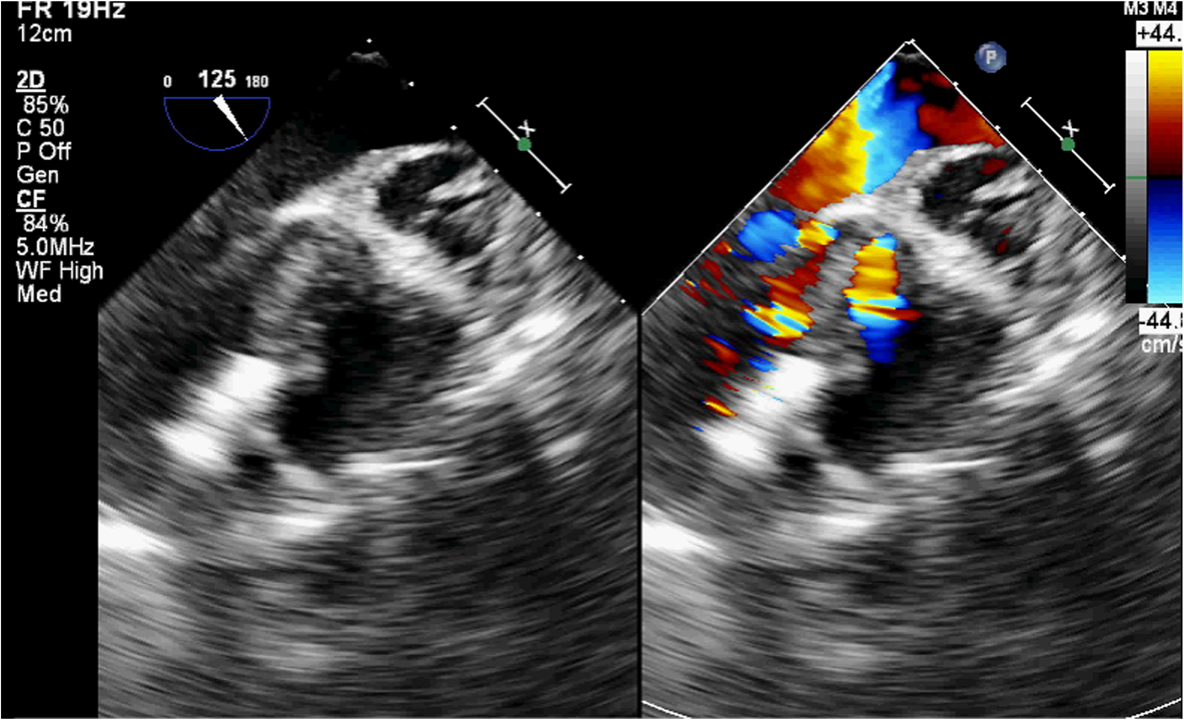
Intraoperative TEE showed that the occluder device on the left ventricular side of the interventricular septum was opened

**Fig. 5:**
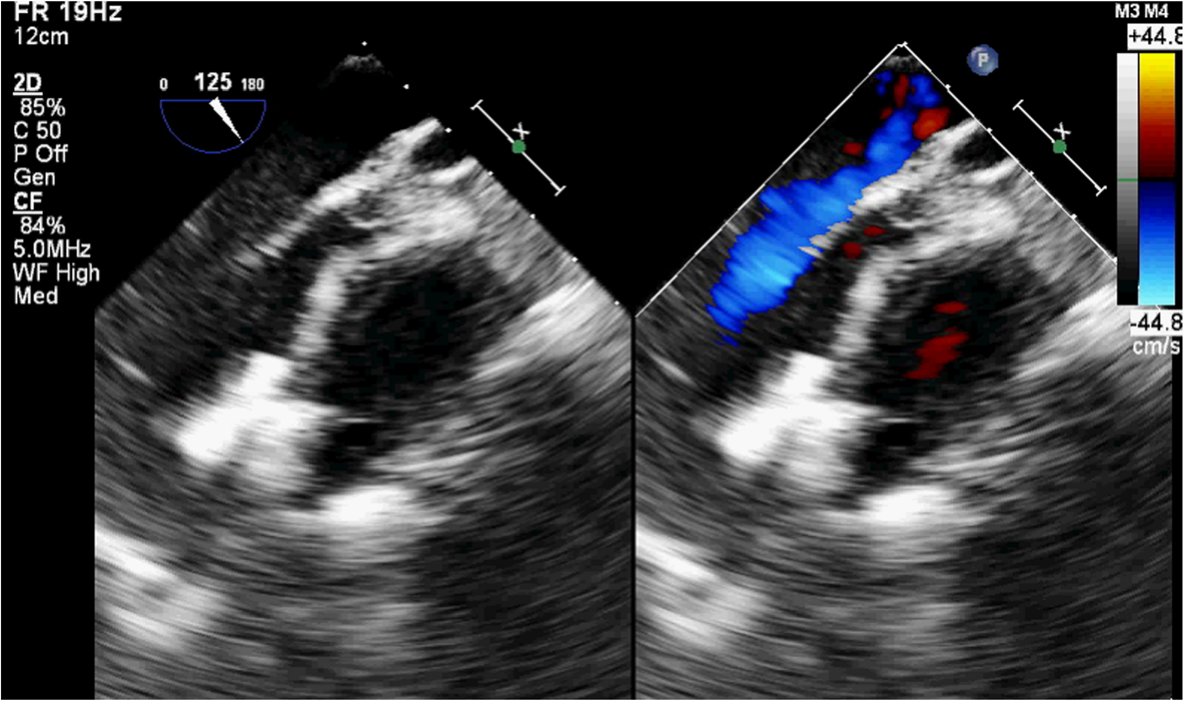
Intraoperative TEE showed that the occluder device was opened on both the left and right ventricular sides at the rupture of interventricular septum

**Fig. 6:**
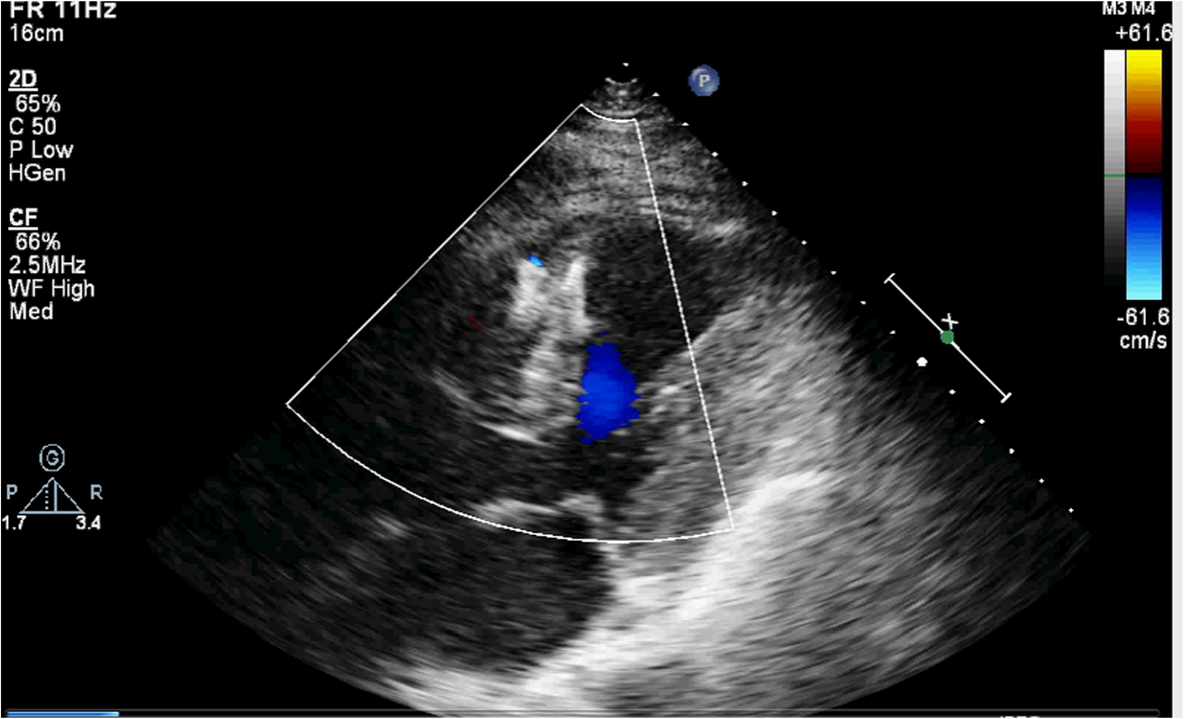
Postoperative reexamination: Occluder device was in normal position

A 77-year-old male patient had a history of myocardial infarction for half a year, but without significant discomfort, lived a normal life at home. Two hours before seeing a doctor, he suddenly developed significant palpitations and shortness of breath and was admitted to the Second Hospital of Lanzhou University by ambulance. The patient’s expression was painful and a distinct murmur was heard in the precordium. Electrocardiogram showed old left ventricular anteroseptal myocardial infarction. X-ray showed enlarged heart shadow. Myocardial enzymes and other laboratory test results showed no significant abnormalities. Echocardiography showed that the interventricular septum was thinned near the apex and bulged toward the right ventricular side, exhibiting “paradoxical motion,” and a rupture opening of about 8 mm in diameter was visible on its top (Fig. 1). CDFI: left ventricular blood shunted to right ventricle through rupture (Fig. 2), the shunt velocity measured by CW is about 410 cm/s, PG:67 mmHg. Left ventricular enlargement (anteroposterior diameter about 61 mm), significantly reduced wall motion amplitude, left ventricular ejection fraction (EF) about 33%, moderate aortic and mitral regurgitation, pulmonary artery systolic pressure about 60 mmHg. Echocardiographic diagnosis: old myocardial infarction of ventricular septal apex with ventricular septal rupture. Due to the patient’s older age and reduced left ventricular function, surgical repair of the site of ventricular septal rupture was very difficult. After multidisciplinary discussion, it was agreed that the patient could not undergo thoracotomy and was not suitable for thoracotomy, and echocardiography guided interventional occlusion of the ruptured interventricular septum could be performed. Interventional occlusion was performed after obtaining the patient’s family’s consent and signing a written consent. Under the guidance of transesophageal echocardiography, the ventricular septal rupture was clearly displayed during the surgery. The size of the rupture was measured again. In order to prevent the occluder device from falling off, a VSD occluder device that is larger and can cover the normal myocardial tissue was selected. Under direct vision, the guide wire and sheath smoothly passed through the ventricular septal rupture (Fig. 3). The left ventricular side of the occluder device was opened and the occluder device was gently pulled to determine the stability of the occluder device (Fig. 4). Then the right ventricular side of the occluder device was opened (Fig. 5), and then the occluder device was gently pushed to determine the stability of the occluder device again to ensure that the occluder device was stable and there was no residual shunt around the occluder device. The operation was completed. After repeated examinations within one year, the occluder device was in normal position without residual shunt(Fig. 6), left ventricular diameter was gradually decreased (anteroposterior diameter was about 52 mm), left ventricular function was improved, and left ventricular EF reached 46%. The patient can live a normal life.

## Discussion

Myocardial infarction with ventricular septal perforation will cause rapid hemodynamic changes, a serious threat to the patient’s life, often died shortly after the onset [1]. Twenty-five percent of patients died within 24 h of onset, 50% died within 1 week, and 70% died within 2 weeks [2]. Only 20% survive more than 1 month after onset [3]. The only treatment is to perform surgery to repair the rupture and correct the hemodynamic disturbance [4]. The current preference for surgical treatment is to treat it differently, depending on the condition. In some cases, no severe hemodynamic changes were produced after ventricular septal perforation, as shown by no significant reduction in cardiac output, no signs of cardiogenic shock, no symptoms of increased pulmonary venous pressure, good renal function, no reduction in urine output, and normal renal function. The patient can closely observe the condition and continue medical treatment. For most cases of ventricular septal perforation, surgery should be performed as soon as possible due to rapid changes in hemodynamics after the onset of the disease, rapid deterioration of circulatory system function, presentation of cardiogenic shock, increased pulmonary venous pressure, decreased renal function, fluid imbalance and other symptoms. Although the early operative mortality after ventricular septal perforation is high, only with surgical treatment, it is possible to save the lives of a proportion of patients. In this case, the patient was unable to undergo thoracotomy, and echocardiography guided interventional closure of the ventricular septal rupture was used, and the entire procedure was uneventful. The patient was followed up for one year, and the closure effect was good, and the patient’s life was stable. This case illustrates that echocardiography guided interventional occlusion may be an alternative method for patients with myocardial infarction and ventricular septal rupture.

## Conclusion

Transesophageal echocardiography-guided interventional occlusion of myocardial infarction with ventricular septal rupture in elderly patients with cardiac insufficiency is a valuable attempt, the successful treatment of this case shows that this method is feasible, for some patients is an appropriate treatment.

## Data Availability

Please contact authors for data requests.

## Abbreviations

CDFI: Color Doppler Flow Imaging
CW: Continuous Doppler
EF: Ejection Fraction
PG: Pressure Gradient
TEE: Transesophageal Echocardiography
TTE: Transthoracic Echocardiogram
VSD: Ventricular Septal Defect
